# Engineered Exosomes in Precision Neuro-Oncology: Mechanisms, Therapeutics, and Translational Challenges

**DOI:** 10.3390/cancers18121923

**Published:** 2026-06-12

**Authors:** Nazmul H. Khan, Mst Anika Bushra, Fowzia Akter Selina, Ali Syed Arbab

**Affiliations:** Georgia Cancer Center, Augusta University, Augusta, GA 30912, USA

**Keywords:** brain diseases, exosomes, therapeutic payloads, blood–brain barrier

## Abstract

Exosomes are small vesicles released by cells that have attracted growing interest as drug delivery vehicles, particularly for brain diseases, where getting therapeutics across the BBB remains a fundamental problem. While conventional platforms such as liposomes, polymeric nanoparticles, and viral vectors often suffer from immune clearance and poor brain accumulation, engineered exosomes leverage natural cellular transport mechanisms to cross the BBB, protect cargo from degradation, and enable biocompatible interactions with target cells. This review takes a mechanistic and translational look at how exosomes are being engineered for CNS disorders, with a particular focus on glioblastoma. We cover exosome biogenesis through ESCRT-dependent and ESCRT-independent pathways, and how the competition between Rab27-driven secretion and Rab7-driven lysosomal degradation determines how many exosomes a cell releases, which has direct consequences for therapeutic production. We then discuss cargo loading strategies, from genetic approaches where donor cells are engineered to package specific molecules during biogenesis to physical methods like electroporation and sonication applied to isolated vesicles, alongside surface modification techniques for directing exosomes toward specific cell types. In glioblastoma, engineered exosomes have shown real promise for delivering chemotherapeutics across the BBB, targeting glioma stem cells, enabling CRISPR-based gene editing, and functioning as combined treatment and imaging tools. Applications in stroke and neurodegenerative diseases, where engineered exosomes carrying microRNAs and neuroprotective cargo have produced encouraging preclinical results, are also discussed. Scalable manufacturing and consistent targeting remain the hardest unsolved problems, and we outline emerging approaches including bioreactor-based production, programmable cargo loading, and patient-specific exosome design that are beginning to address these gaps. Overall, the progress reviewed here suggests that engineered exosomes are moving from an interesting biological concept toward a practically viable platform for CNS drug delivery.

## Simple Summary

Brain tumors and other central nervous system (CNS) diseases remain difficult to treat because the blood–brain barrier (BBB) limits the delivery of most therapeutic agents into the brain. Engineered exosomes have emerged as promising natural nano-sized delivery vehicles capable of crossing the BBB while carrying therapeutic molecules such as drugs, RNA, proteins, and gene-editing systems. Recent advances have improved how these vesicles are loaded with therapeutic cargo, directed toward tumor cells, and tracked in real time. This review summarizes the molecular mechanisms governing exosome biogenesis, BBB transport, cargo engineering, and tumor targeting, with particular emphasis on glioblastoma, and discusses combined treatment-and-imaging platforms, scalable manufacturing, purification technologies, donor-cell selection, and translational barriers that must be overcome before clinical use. By integrating mechanistic biology with engineering and translational perspectives, this review provides a practical framework for developing precision exosome therapeutics for brain cancer and other CNS diseases.

## 1. Introduction

Central nervous system (CNS) disorders, including glioblastoma, stroke, Alzheimer’s disease, and Parkinson’s disease, remain difficult to treat effectively despite extensive therapeutic development [1,2,3,4]. A major barrier to clinical success is the limited brain bioavailability of therapeutics, due to the highly restrictive nature of the blood–brain barrier (BBB). Formed by tightly connected endothelial cells and supported by astrocytes, pericytes, and the basement membrane, the BBB prevents most small molecules and nearly all biologics from entering the brain. Conventional delivery systems (liposomes, polymeric nanoparticles, and viral vectors) have shown limited success in overcoming the BBB, as detailed in Section 2, underscoring the need for biologically inspired alternatives [5,6,7,8,9,10,11,12]. These combined limitations contribute to the high failure rate of CNS-targeted therapies in clinical trials and highlight the urgent need for more effective brain delivery strategies. Among emerging alternatives, exosomes have attracted particular interest due to their intrinsic biocompatibility, stability in circulation, and natural capacity to transport bioactive cargo across biological barriers, including the BBB. Their ability to protect molecular payloads, interact with recipient cells, and modulate cellular function positions them as a compelling platform for brain-targeted diagnostics and therapeutics [13,14].

Several recent publications have highlighted the therapeutic potential of exosomes in brain diseases and cancer, including their roles in drug delivery, tumor biology, and biomarker development [15,16,17,18,19]. To fully appreciate this landscape, it is necessary to consider how these platforms sit within recent literature. For instance, Jin et al. provided a detailed summary of engineered exosomes as precision tools for brain-targeted delivery, focusing heavily on baseline biomaterials and general central nervous system diseases [20]. Similarly, Johnston et al. outlined the custom manufacturing of extracellular vesicle therapeutics tailored for neurological conditions, highlighting the general clinical scaling pathways [21]. Lee et al. explored the broad dualities of advances and challenges in central nervous system disorders [22], while a comprehensive review in the same year systematically classified extracellular vesicles acting as drug and gene delivery vehicles across diverse brain injuries [23]. Rather than duplicating these broad or general overviews, the present review aims to provide a more integrated and mechanistic perspective on engineered exosomes in neuro-oncology. In particular, we emphasize (1) a detailed examination of exosome biogenesis and secretion pathways, including Endosomal Sorting Complex Required for Transport (ESCRT)-dependent and ESCRT-independent mechanisms and their regulatory balance with lysosomal degradation; (2) a systematic analysis of engineering strategies, with a focus on cargo loading approaches and surface functionalization for targeted delivery; and (3) the emerging role of engineered exosomes in theragnostic applications, especially in glioblastoma. By connecting these mechanistic insights with translational challenges and disease-specific applications, this review seeks to clarify how advances in exosome biology can be leveraged to improve precision delivery and therapeutic outcomes in central nervous system disorders.

## 2. Biological Barriers to Brain Drug Delivery

### 2.1. The Blood–Brain Barrier (BBB)

Beyond its structural complexity, the BBB presents two challenges that are especially relevant for drug delivery. First, although conditions like glioblastoma or acute stroke can locally disrupt tight junction integrity, the resulting increase in permeability is spatially uneven and unpredictable, so passive leakage alone cannot be relied upon for consistent therapeutic access [24,25,26,27]. Second, even when a drug manages to cross the endothelium, efflux transporters such as P-glycoprotein and breast cancer resistance protein (BCRP) actively pump it back into circulation before it can accumulate in the brain [26]. Together, these two properties explain why the BBB is so difficult to overcome in practice and why simply waiting for disease-related barrier disruption is not a viable delivery strategy.

### 2.2. Endogenous Carriers

The limitations of synthetic carriers highlight the need for alternative delivery systems that leverage natural cellular pathways [28]. However, even endogenous carriers like exosomes must contend with significant biological obstacles [29]. In circulation, exosomes are subject to rapid clearance by the mononuclear phagocyte system, particularly uptake by macrophages in the liver and spleen, which substantially reduces the fraction that reaches the brain [30]. Surface opsonization by serum proteins accelerates this clearance further by enabling recognition by macrophage cell-surface receptors [29]. Once in the CNS, exosomes must still navigate the extracellular matrix and the steep concentration gradients created by the interstitial fluid pressure within tumors, which can limit deep tissue penetration [28]. Additionally, endogenous exosomes lack inherent targeting specificity, meaning that without engineering, they distribute broadly rather than accumulating at disease sites [29,30]. Despite these barriers, exosomes hold a fundamental advantage over synthetic nanocarriers. Unlike conventional synthetic platforms, exosomes exploit natural endogenous trafficking pathways, including receptor-mediated endocytosis and active endothelial transcytosis, which allows them to bypass immune recognition and avoid rapid clearance by systemic phagocytes [30,31,32,33]. The biological basis for these advantages, and how engineering strategies can address the barriers described above, is discussed in detail in Section 6.

## 3. Exosomes as Natural Nanocarriers

**Figure 1:**
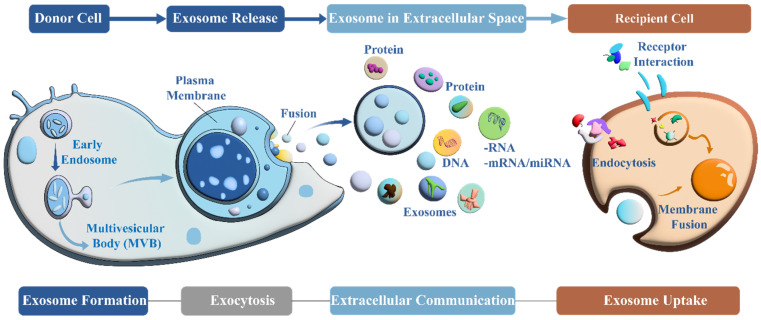
The Lifecycle and Communication Pathway of Exosomes. Schematic representation of exosome biogenesis, secretion, and recipient cell uptake. The process begins within the donor cell where inward budding of early endosomes leads to the formation of Multivesicular Bodies (MVBs). Upon fusion with the plasma membrane (Exocytosis), vesicles are released into the extracellular space as exosomes carrying diverse cargo (Proteins, DNA, RNA). Communication is completed via exosome uptake by the recipient cell through receptor interaction, endocytosis, or membrane fusion.

A schematic overview of exosome biogenesis, degradation, and functional rationale is presented in Figure 1.

### 3.1. Definition and Discovery

Exosomes are small extracellular vesicles (EVs) of endosomal origin, typically 30–150 nm in diameter, produced by most eukaryotic cells and released into the extracellular milieu when intraluminal vesicles (ILVs) within multivesicular bodies (MVBs) are secreted by fusion with the plasma membrane [34]. They contain a complex cargo of proteins, lipids, and nucleic acids that reflect the physiological state of the cell of origin and can be transferred to recipient cells to modulate their function [35,36,37,38]. Although vesicle-like particles were first observed in the 1940s in studies of blood coagulation, they were initially considered cellular debris with limited biological significance [39]. The first experimental evidence of this novel secretory process emerged in the early 1980s during studies of reticulocyte maturation. Using electron microscopy and biochemical tracking of transferrin receptors, Harding and colleagues demonstrated that small vesicles formed inside multivesicular endosomes are externalized into the extracellular space as maturing reticulocytes lose redundant membrane components, establishing the existence of an endosomal exocytosis mechanism [40]. In parallel, work by Johnstone and co-workers showed that these small extracellular secreted vesicles contained specific plasma membrane activities that diminished as reticulocytes matured, providing functional evidence that this was a regulated biological process rather than random membrane shedding [41,42]. Subsequent studies in the 1990s revealed that exosomes secreted by immune cells could participate in antigen presentation, challenging the idea that exosomes were merely cellular waste [43]. In the 2000s, landmark work demonstrated that exosomes carry functional RNA and proteins that can be transferred between cells, establishing them as critical mediators of intercellular communication [36,44]. A growing body of experimental work has since shown that exosomes play crucial roles in physiological and pathological intercellular communication, including immune modulation and transfer of bioactive molecules [45,46]. In translational research, engineered exosomes have been developed to deliver therapeutic cargo such as proteins and RNA to target tissues, highlighting their potential as natural nanocarriers [47,48,49,50].

### 3.2. Biogenesis and Secretion

Exosome biogenesis is a multistep, tightly regulated intracellular process that begins with the formation of intraluminal vesicles (ILVs) within late endosomes, generating multivesicular bodies (MVBs) [37,51,52].

ILV formation can be driven by canonical endosomal sorting complex required for transport (ESCRT) machinery and its accessory factors such as TSG101 and ALIX, which recruit specific cargoes into budding vesicles and facilitate membrane scission [53,54]. Experimental depletion of key ESCRT components (e.g., HRS/ESCRT-0 or TSG101) significantly reduces exosome output, underlining the contribution of ESCRT-dependent sorting to exosome formation [55]. In parallel, non-ESCRT pathways exist: for example, Rab31 facilitates an ESCRT-independent biogenesis route by engaging lipid raft proteins to drive ILV formation; crucially, it simultaneously suppresses MVB-lysosome fusion, thereby shunting MVBs toward the secretory pathway [52].

Once ILVs are established, MVBs undergo maturation and intracellular trafficking along the cytoskeleton. Rab family GTPases are central regulators here: Rab27a/b mediates transport of mature secretory MVBs to the plasma membrane and docking at the cell periphery, while additional small GTPases such as Rab11 in certain contexts contribute to trafficking steps under specific stimuli [56,57]. Upstream cellular signals profoundly influence these pathways. For instance, calcium elevations in cancer cells activate Munc13-4, which interacts with Rab27 and the SNARE fusion machinery (syntaxin-4, SNAP-23, VAMP-7) to enhance MVB docking and fusion at the plasma membrane, increasing exosome release [57,58]. Other stimuli, such as receptor tyrosine kinase signaling pathways, can modulate exosome secretion by activating small GTPases like Rab31 [52].

### 3.3. Exosome Release and Degradation

**Figure 2:**
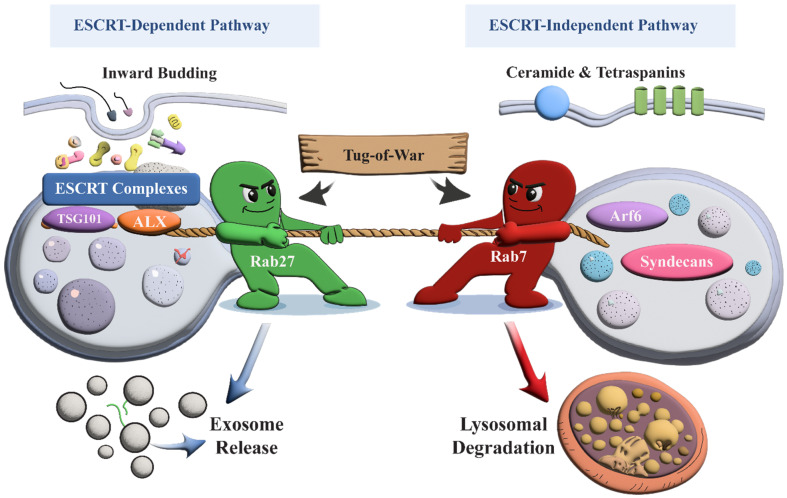
Competing pathways governing MVB fate: exosome secretion versus lysosomal degradation. The fate of multivesicular bodies (MVBs) is determined by a competitive balance between Rab27-mediated secretion ((**left**), green) and Rab7-dependent lysosomal degradation ((**right**), red), as described in Section 3.2 and Section 3.3. In the ESCRT-dependent pathway (**left**), sequential recruitment of ESCRT complexes and accessory proteins drives inward budding and ILV formation; when Rab27 dominates, MVBs fuse with the plasma membrane and release exosomes. In the ESCRT-independent pathway (**right**), ceramide, tetraspanins, Arf6, and syndecans mediate ILV biogenesis; when Rab7 dominates, MVBs are routed to lysosomes for degradation. The ESCRT-dependent and ESCRT-independent pathways represent alternative mechanisms of ILV biogenesis and are not intended to imply exclusive routing toward secretion or degradation. Rather, the ultimate fate of MVBs is determined primarily by the balance between Rab27- and Rab7-mediated trafficking. The relative activity of these two GTPases acts as a regulatory switch controlling the quantity and composition of secreted exosomes. Understanding this regulatory balance is directly relevant to therapeutic production, as strategies that favor Rab27-mediated secretion over Rab7-dependent degradation can increase exosome yield from engineered donor cells, a point taken up in Section 7.

After transport, MVBs fuse with the plasma membrane via coordinated action of tethering factors, SNARE (Soluble N-ethylmaleimide-sensitive factor Attachment Protein Receptor) complexes, and Rab effectors, enabling ILV discharge as exosomes [58,59]. In contrast, MVBs can also be routed to lysosomal degradation, regulating the turnover of both membranes and cargo. Factors that bias MVBs toward the degradative pathway include active Rab7 and posttranslational modifications that promote lysosome fusion, reducing exosome release [60,61]. The balance between secretory and degradative pathways is tightly regulated and determines the stability, quantity, and functional roles of exosomes in both normal and disease states [52,56,59]. The fate of an MVB is determined by a competitive “molecular tug-of-war” between Rab27-mediated secretion and Rab7-dependent lysosomal degradation (Figure 2), a critical regulatory node that dictates the overall rate of exosome production.

## 4. Engineering Exosomes for Therapeutics

Although native exosomes transport proteins, lipids, and nucleic acids between cells, their inherent cargo composition and surface properties are rarely optimal for therapeutic applications. Bioengineering strategies have therefore been developed to enhance cargo loading, improve tissue targeting, and enable controlled delivery, either by modifying the parental cells that produce exosomes or by directly altering purified vesicles after isolation [62,63].

### 4.1. Cargo Loading

Efficient incorporation of therapeutic cargo is the central aspect of exosome engineering. Loading strategies can be broadly divided into endogenous loading, in which donor cells are genetically modified to package specific molecules into exosomes during biogenesis [64], and exogenous loading, in which purified vesicles are directly loaded with therapeutic compounds after isolation [65].

**Figure 3:**
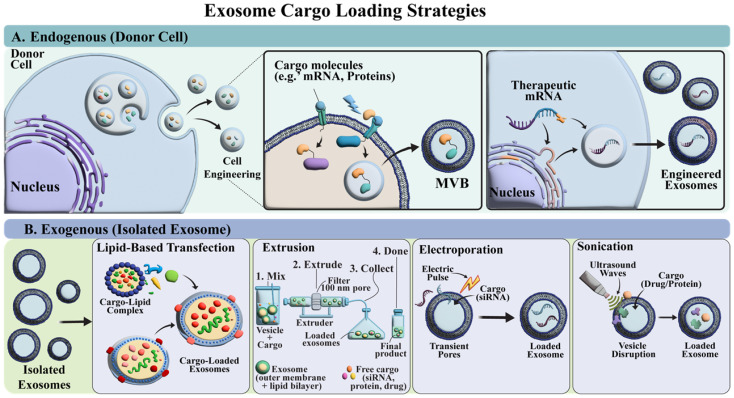
Strategies for cargo loading into exosomes. (**A**) Endogenous loading through genetic engineering of donor cells (e.g., EXPLOR or EXOtic systems), enabling incorporation of therapeutic proteins or mRNA during biogenesis within multivesicular bodies (MVBs). (**B**) Exogenous loading of isolated exosomes using physical or chemical methods, including lipid-based transfection, extrusion through polycarbonate membranes, electroporation, and sonication. See Section 4.1 for detailed mechanisms, advantages, and limitations of each approach.

In endogenous loading approaches (Figure 3A), donor cells are engineered to express therapeutic proteins or nucleic acids that are selectively sorted into intraluminal vesicles during multivesicular body formation, allowing the desired cargo to be incorporated into exosomes during biogenesis [66,67,68,69]. For example, the EXPLOR (EXosomal Loading via Optically Reversible protein–protein interactions) system enables controlled protein loading by fusing cargo proteins to Cryptochrome 2 (CRY2) and exosomal membrane proteins to the N-terminal fragment of Cryptochrome-Interacting Basic-helix-loop-helix 1 (CIBN) [66,70]. Upon blue light stimulation, CRY2-fused proteins are recruited to exosome-forming membranes and incorporated into intraluminal vesicles [67,70]. Exosomes produced via EXPLOR efficiently contained functional proteins, demonstrating that intracellular engineering of donor cells can precisely control protein incorporation during exosome biogenesis [66,67]. Similar strategies have been applied to nucleic acids, where donor cells engineered to express therapeutic mRNA produce exosomes that encapsulate and transfer functional transcripts to recipient cells. For example, genetic devices termed EXOtic have been developed to enhance selective mRNA recruitment into exosomal membranes, enabling engineered exosomes to deliver functional mRNA such as catalase transcripts and induce protein expression in recipient cells, highlighting the feasibility of exosome-mediated nucleic acid delivery for therapeutic applications [71].

Exogenous loading methods (Figure 3B) allow therapeutic molecules to be incorporated into isolated exosomes using physical or chemical approaches. Electroporation, one of the most widely used techniques, transiently permeabilizes the exosomal membrane, enabling nucleic acids such as siRNA or microRNA to enter the vesicle lumen. However, its efficiency remains debated, as studies have shown that electroporation can induce siRNA aggregation and lead to overestimation of loading efficiency, highlighting the need for careful optimization and validation of this approach [65]. Other strategies for cargo incorporation include sonication, extrusion, and chemical transfection reagents, which facilitate loading of small molecules or proteins while preserving vesicle integrity. Sonication has been used to encapsulate the chemotherapeutic drug paclitaxel into exosomes, resulting in higher drug loading and improved cancer cell killing relative to free drug [72]. Methods such as sonication and extrusion have been used to incorporate the therapeutic protein catalase into exosomes or exosome-mimetic vesicles, demonstrating effective cargo encapsulation and delivery [73]. Lipid-based transfection approaches can form complexes with RNA or RNA/DNA nanostructures and facilitate their incorporation into isolated exosomes while maintaining vesicle stability [74]. Efficient cargo loading remains a critical determinant of the therapeutic efficacy of engineered exosomes, and ongoing research continues to optimize these strategies while preserving exosome integrity and biological activity.

### 4.2. Surface Targeting

Beyond cargo loading, exosomal surfaces must be engineered to direct vesicles toward specific tissues or cell types, enabling targeted therapeutic delivery. This surface targeting is commonly achieved either by genetically modifying donor cells [64] or by chemically conjugating targeting ligands onto purified exosomes [75].

A widely used strategy for exosome targeting involves displaying peptides on the exosomal surface by fusing them to membrane proteins such as Lamp2b [64,76]. Donor cells are engineered to express a Lamp2b-targeting ligand fusion, which is incorporated into exosomes during biogenesis, presenting the peptide on the surface [64,77]. This allows engineered exosomes to bind specific receptors on recipient cells, enabling targeted cargo delivery. A notable example is the rabies virus glycoprotein (RVG) peptide, which binds neuronal acetylcholine receptors and facilitates transport across the BBB. RVG-modified exosomes have been shown to deliver therapeutic cargo to neurons in vivo, achieving efficient brain targeting following systemic administration [64,78]. Extending this concept beyond neuronal targeting, engineered exosomes have also been designed to selectively recognize immune cell populations within the tumor microenvironment using specific peptide fusions [79,80]. In one approach, genetically modified HEK293 cells were used to produce exosomes displaying a CD206-targeting peptide fused to an exosomal membrane protein, enabling specific binding to CD206-positive M2 macrophages [79,81]. These engineered vesicles accumulated at tumor and metastatic sites in vivo and could be further functionalized with an Fc domain to promote antibody-dependent cellular cytotoxicity (ADCC) [79]. CRISPR-Cas9-loaded exosomes have been investigated, leading to depletion of protumorigenic macrophages and reduced tumor burden. This work illustrates how surface-engineering strategies originally developed for targeted delivery can be adapted to modulate disease-associated cell populations, expanding the therapeutic scope of exosome-based platforms beyond cargo transport toward active remodeling of the tumor microenvironment [79]. Our laboratory has extensively used Lamp2b to display targeting peptides against multiple cell populations, including neutrophils, GBM cells, and neurons, and to incorporate therapeutic payloads such as neuroglobin (Ngb) [79,80].

**Figure 4:**
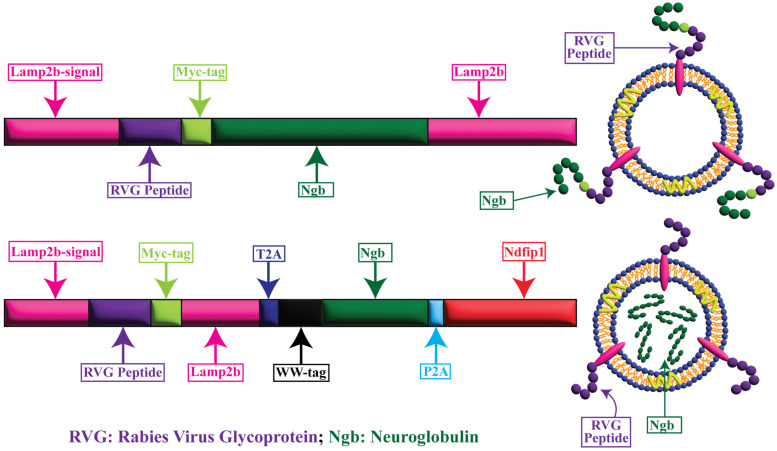
Genetic engineering of exosomes for brain-targeted delivery of neuroglobin (Ngb) [80]. Schematic representation of two construct designs for surface-targeted, Ngb-loaded exosomes, as described in Section 4.2. (**Top**) Surface-displayed Ngb construct. The first-generation construct encodes a linear fusion protein comprising, from N- to C-terminus: a Lamp2b signal sequence (magenta), an RVG peptide (purple) for neuronal targeting via acetylcholine receptor binding, a Myc epitope tag (yellow green) for detection, and the full-length Lamp2b transmembrane domain (green/magenta). Ngb (dark green) is fused within the extracellular region of Lamp2b, resulting in its display on the outer surface of secreted exosomes. The corresponding exosome schematic (right) shows RVG peptides decorating the surface alongside externally displayed Ngb clusters, with Lamp2b anchoring the fusion protein in the lipid bilayer. (**Bottom**) Lumen-loaded Ngb construct. The second-generation construct incorporates an Ndfip1 domain (red) to redirect Ngb into the exosome lumen. The construct encodes: Lamp2b signal sequence, RVG peptide, Myc-tag, and Lamp2b transmembrane domain, followed by a T2A self-cleaving peptide sequence (dark blue) and Ngb linked via a P2A element (cyan) to Ndfip1. The self-cleaving sequences allow co-translational separation of the Lamp2b/RVG surface component from the Ngb/Ndfip1 cargo module, which is sorted into the vesicle interior during MVB formation. The corresponding exosome schematic (right) shows RVG retained on the exosomal surface while Ngb is packaged within the lumen. The two designs differ in Ngb localization (surface-displayed versus lumen-encapsulated), while both retain RVG-mediated neuronal targeting via the Lamp2b fusion architecture. RVG: Rabies Virus Glycoprotein. Ngb: Neuroglobin.

Our laboratory has also developed a platform that utilizes genetically modified HEK293 cells to produce engineered exosomes carrying RVG on the surface of the exosomes and the payload (neuroglobin, Ngb) either on the surface or inside the lumen of the exosomes (Figure 4). We have shown effective accumulation of the exosomes in the brain, and there was significant improvement in the strokes outcomes following treatments with engineered exosomes compared to that of control HEK293-derived exosomes [80].

More recent studies have extended exosome targeting strategies using chemical conjugation. In one approach, RVG peptides were attached to the surface of exosomes using bio-orthogonal click chemistry, creating vesicles capable of specifically targeting neurons. These engineered exosomes were loaded with the neuroprotective peptide NR2B9c and administered intravenously in a mouse model of traumatic brain injury. Compared with non-targeted exosomes, the RVG-modified exosomes delivered NR2B9c more efficiently to neurons, reduced neuronal damage, and improved functional recovery, demonstrating the potential of chemical surface engineering for targeted therapeutic delivery [78].

In addition to peptide targeting, antibodies, aptamers, and other receptor-binding ligands can be incorporated onto exosome surfaces to enhance delivery to tumor cells or inflamed tissues. These surface modifications allow engineered exosomes to exploit receptor-mediated uptake pathways, increasing delivery efficiency to specific cell populations [75,82,83]. For example, the DNA aptamer sgc8 can be chemically conjugated to a lipid moiety and incorporated onto exosomes, enabling them to specifically target CCRF-CEM cells, a human T cell acute lymphoblastic leukemia (T-ALL) line, by binding to protein tyrosine kinase 7 (PTK7) expressed on their surface [82]. Similarly, monoclonal antibodies can be displayed on exosome surfaces to direct them toward tumor cells. In one study, exosomes decorated with antibodies were loaded with the anticancer drug romidepsin, resulting in enhanced tumor targeting and improved delivery through receptor-mediated uptake [83]. Table 1 summarizes the major engineering strategies for exosome cargo loading and surface targeting, including their mechanisms, advantages, and limitations.

### 4.3. Therapeutic Payloads

The suitability of a given cargo for exosome-mediated delivery depends not only on the loading method used but on the intrinsic biochemical properties of the molecule itself. Understanding these properties is important for matching cargo to the right loading strategy and anticipating stability and release challenges in vivo.

Nucleic acid therapeutics, including siRNA, miRNA, mRNA, and CRISPR components, are among the most actively explored exosome payloads, but they present significant delivery challenges [65,84]. As negatively charged macromolecules, they do not passively cross lipid bilayers, making active loading methods such as electroporation or endogenous sorting essential. Once inside the lumen, the lipid bilayer provides meaningful protection against serum nucleases that would otherwise degrade naked RNA within minutes of systemic administration. However, loading efficiency varies considerably by method and RNA species, and aggregation during electroporation remains a recognized problem that can overestimate apparent encapsulation [65]. mRNA presents additional challenges due to its size and susceptibility to degradation, making endogenous loading via engineered donor cells generally more reliable than post-isolation methods for functional mRNA delivery [71].

Protein cargo requires careful consideration of size, folding state, and whether surface display or luminal encapsulation is more appropriate for the intended application. Small proteins and peptides can be loaded post-isolation via sonication or extrusion [72,73], but larger or structurally sensitive proteins are better incorporated endogenously to preserve their functional conformation [66]. Proteins destined for luminal encapsulation benefit from fusion to sorting signals such as Ndfip1, which directs cargo into the MVB pathway during biogenesis, as demonstrated in the Ngb loading platform described in Section 4.2 [80]. Surface-displayed proteins, by contrast, require fusion to transmembrane anchors such as Lamp2b and are exposed to the extracellular environment, which affects both targeting function and potential immunogenicity [64,79,80].

Small-molecule drugs behave quite differently from biologics in the context of exosome loading. Hydrophobic molecules partition naturally into the lipid bilayer during simple incubation, making passive loading practical for many chemotherapeutic agents [72,85]. Small Hydrophilic molecules, however, require active loading methods to achieve meaningful luminal concentrations [65,85]. The lipid bilayer protects encapsulated drugs from premature metabolism and reduces systemic exposure, improving the therapeutic index relative to free drug administration [72,73]. Drug release kinetics depend on membrane composition and the physicochemical properties of the molecule, and these parameters can be tuned to some extent by modifying the lipid composition of donor cells or by using hybrid exosome–liposome platforms that offer greater control over encapsulation and release [86,87]. The disease-specific applications of these payload classes in glioblastoma, stroke, and neurodegeneration are discussed in detail in Section 5.

### 4.4. Choosing the Right Cell Factory: Classification and Selection of Donor Cells

The therapeutic potential of an engineered exosome depends largely on its parental donor cell, which influences membrane composition, cargo loading capacity, biological activity, and manufacturing scalability. In neuro-oncology, three donor cell sources are used most frequently, each offering distinct advantages [64,83,88].

Mesenchymal stem or stromal cells (MSCs) are widely favored because of their natural tropism toward glioblastoma-associated inflammation. MSC-derived exosomes are highly biocompatible, can help stabilize the blood–brain barrier (BBB), and efficiently deliver therapeutic microRNAs such as miR-124 and miR-133b while largely avoiding immune clearance [89,90,91].

Human embryonic kidney (HEK293) cells are commonly used as manufacturing platforms due to their scalability and ease of genetic engineering. These cells are particularly useful for producing targeted exosomes, such as those displaying rabies virus glycoprotein (RVG) peptides that facilitate BBB crossing and brain-specific delivery [64,92,93].

Immune-cell-derived exosomes, including those from neural stem cells, macrophages, and natural killer cells, offer additional therapeutic benefits. Unlike tumor-derived exosomes, which often promote immune evasion, engineered immune-cell exosomes can help reprogram the immunosuppressive glioblastoma microenvironment and enhance antitumor responses [94,95,96,97].

The choice of donor cell also has important translational implications. Animal-derived cells are useful for preclinical studies, but present challenges related to immunogenicity and pathogen transmission. Human-derived immortalized cell lines such as HEK293 reduce these concerns but require stringent purification to remove residual host-cell proteins and DNA [98]. Consequently, clinical manufacturing typically incorporates purification strategies such as tangential flow filtration (TFF) and size-exclusion chromatography (SEC) [99,100].

Rather than a single optimal source, donor-cell selection should be guided by the intended therapeutic goal, whether it involves RNA delivery, advanced targeting strategies, or modulation of the tumor microenvironment [64,89,96,97].

## 5. Exosomes in CNS Diseases

With the engineering framework established in Section 4, we now turn to how these strategies have been applied across specific CNS disease contexts. The discussion is organized around brain tumors, stroke, and neurodegeneration, with glioblastoma receiving the most detailed treatment given the depth of available preclinical and translational data.

### 5.1. Exosomes in Brain Tumors

Brain tumors, and glioblastoma in particular, are among the most challenging cancers to treat because therapeutic agents struggle to penetrate the BBB and accumulate in sufficient amounts at the tumor site. Exosomes, with their natural ability to cross the BBB and deliver biologically active cargo, have emerged as an important nanoplatform for both understanding glioma biology and developing targeted therapies [15].

#### 5.1.1. The Role of Exosomes on Glioblastoma

In brain tumors, including glioblastoma, astrocytoma, meningioma, and medulloblastoma, tumor-derived exosomes significantly influence tumor progression, invasion, angiogenesis, immune evasion, and therapy resistance [15,101]. Glioblastoma cells release abundant exosomes that carry oncogenic proteins, microRNAs, and other factors that contribute to tumor growth, invasion, neovascularization, and resistance to therapy. These vesicles can remodel the tumor microenvironment, influence immune responses, and help tumor cells adapt to stress [101]. These vesicles carry a rich cargo of proteins (e.g., EGFRvIII, ANXA1, B7-H3), nucleic acids (miRNAs such as miR-21, lncRNAs, circRNAs), and lipids that modulate signaling pathways in recipient cells, promoting proliferation, neovascularization, and metabolic reprogramming [102,103,104,105,106]. Beyond glioblastoma, exosomes derived from metastatic tumors (e.g., lung and breast cancer metastases) breach the intact BBB through transcytosis and LCN2-mediated mechanisms, establishing a permissive pre-metastatic niche [103,107]. Their cargo often reflects tumor subtype-specific signatures, including transcription factors, membrane proteins, and microRNAs, which can serve as valuable diagnostic and prognostic biomarkers [92,108]. Exosomes also contribute to immune evasion, for instance by inducing ferroptosis in dendritic cells via the NRF2/GPX4 pathway and driving M2 macrophage polarization [93,109]. This dual role in pathogenesis and liquid biopsy underscores their relevance to both diagnosis and therapy [102,108].

##### Delivery of Therapeutic Molecules

The intrinsic ability of exosomes to cross the BBB makes them highly promising vehicles for delivering therapeutic cargo to brain tumors. Tumor-derived and engineered exosomes efficiently transport small molecules, antibodies, RNA therapeutics, and genome-editing components into the CNS [85,101,107]. Exosomes derived from C6 glioma cells have been engineered to carry a dual therapeutic payload combining surface-displayed cetuximab with intraluminal doxorubicin. Cetuximab was attached to the exosome surface via a PEG-lipid insertion method, positioning the antibody outward to target EGFR on tumor cells, while doxorubicin was incorporated into the lumen through simple incubation, enabling protection during circulation and intracellular release after uptake. This platform showed enhanced BBB penetration, improved tumor cytotoxicity, and significantly prolonged survival in glioma-bearing rats compared with free drugs [110].

Embryonic stem cell-derived exosomes with targeting properties and inflammation-responsive neutrophil-derived exosomes further enhance selective delivery in the tumor microenvironment [111,112]. Beyond chemotherapy, exosomes modified with targeting ligands such as RGD peptides show enhanced accumulation in glioblastoma cells, improving cytotoxicity and reducing off-target effects. Embryonic stem cell-derived exosomes were engineered to carry paclitaxel and a cyclic RGD peptide (c RGDyK) on their surface. The RGD ligand, which is not therapeutic itself, targets integrin receptors on glioblastoma cells and tumor neovasculature, enhancing uptake of the exosomes. This formulation, called cRGD-Exo-PTX, showed higher accumulation in glioblastoma cells in vitro and in mouse models compared with unmodified exosomes or free paclitaxel. It produced stronger tumor fluorescence signals, greater inhibition of tumor growth, increased apoptosis, and longer survival, demonstrating that surface-targeted exosomes can improve delivery of chemotherapeutics while reducing off-target effects [111]. Neutrophil-derived exosomes that respond to inflammatory cues in the tumor microenvironment have been developed as targeted delivery vehicles for glioma therapy. In a glioma model, neutrophil exosome-based carriers loaded with chemotherapeutic agent doxorubicin were shown to penetrate the BBB, migrate toward inflamed tumor tissue, and preferentially release cargo within the glioma microenvironment, resulting in enhanced tumor targeting compared with non-responsive vesicles. This demonstrates that leveraging neutrophil inflammatory responsiveness can improve selective cargo release in glioma treatment [112].

Exosomes isolated from glioblastoma cells themselves can also be loaded with drugs like selumetinib and demonstrate innate tropism toward tumor sites, offering a way to leverage tumor-specific targeting without any external ligands. In their study, Lee and colleagues isolated exosomes from human U87MG glioblastoma cells and loaded them with the anticancer drug selumetinib via electroporation. These glioblastoma-derived exosomes naturally accumulated in glioma cells in vitro and in vivo, demonstrating an innate preference for the parent tumor and its microenvironment. In mouse GBM xenografts, selumetinib delivered this way suppressed tumor growth more effectively than free drug and caused minimal toxicity in the normal brain, liver, or kidney. This work shows that tumor-derived exosomes can leverage their origin for targeted drug delivery without the need for added ligands [113].

At the same time, all these exosome strategies have limitations. Tumor-derived exosomes carry proteins, lipids, and nucleic acids that can actively suppress immune functions. Experimental studies show that exosomes from cancer cells induce suppressive CD8^+^ T cells, inhibit T cell activation and proliferation, and blunt antitumor immunity. They can also interfere with therapeutic antibody binding and reduce antibody-dependent cell-mediated cytotoxicity (ADCC), reshaping the immune environment in ways that favor tumor survival [94,95]. Work examining environmental and molecular contributors to glioblastoma progression has also highlighted signaling pathways that may intersect with exosome biology. For example, glyphosate exposure was shown to target the Src-family kinase FYN, activating PI3K-AKT-mTOR signaling and enhancing glycolysis, proliferation, migration, and invasion in glioblastoma models. Suppression of FYN reversed these effects and reduced exosome-mediated polarization of M2-like macrophages and immunosuppressive factor secretion, suggesting that modulation of metabolic and signaling networks like FYN may influence both tumor cell behavior and the impact of tumor-derived vesicles on the microenvironment [114].

Advances in RNA therapeutics have utilized exosomes for efficient delivery of mRNA, siRNA, and antisense oligonucleotides. Cellular nanoporation techniques enable large-scale generation of functional mRNA-encapsulating exosomes with improved stability and delivery efficiency [88]. T7 peptide-decorated exosomes effectively silence oncogenic miR-21, while plant-derived ginger exosomes demonstrate direct therapeutic efficacy against glioblastoma [115,116]. Combination therapies using membrane-decorated or hybrid exosome–liposome platforms allow co-delivery of chemotherapeutics and nucleic acids, achieving synergistic antitumor effects with reduced off-target toxicity [32,117].

##### Engineered and Theragnostic Exosomes

Beyond simple drug carriage, engineered exosomes are increasingly being developed as multifunctional platforms for combination therapy, gene regulation, and theragnostic applications in glioblastoma [118,119]. In these systems, therapeutic payload delivery is integrated with molecular or imaging components that enable real-time tracking of biodistribution and tumor targeting in the brain, thereby allowing direct correlation between BBB transport efficiency, intracranial pharmacokinetics, and therapeutic response in glioblastoma models [120]. In preclinical glioblastoma studies, co-delivery strategies have shown promise, especially where chemotherapeutic agents are combined with resistance-modulating molecules to overcome intrinsic or acquired drug resistance. For instance, exosome-based co-delivery systems incorporating temozolomide (TMZ) with O^6^-benzylguanine (a potent MGMT inhibitor) have been explored to sensitize glioma cells by suppressing DNA repair pathways, thereby improving TMZ cytotoxicity and reducing chemoresistance-associated tumor recurrence in intracranial glioma models [117]. These strategies are further strengthened by surface-engineering approaches that enhance BBB penetration and tumor selectivity. Functionalization with ligands such as Angiopep-2 enables targeting of low-density lipoprotein receptor-related protein-1 (LRP1), which is highly expressed on BBB endothelial cells and glioma cells, thereby facilitating receptor-mediated transcytosis into the brain tumor microenvironment [121]. In parallel, CD133-targeting aptamers have been incorporated into engineered exosomes to selectively bind glioma stem-like cells, a subpopulation strongly associated with recurrence and therapeutic resistance, thereby improving intratumoral specificity and reducing off-target toxicity in orthotopic glioblastoma models [117].

In addition to ligand-based targeting, exosomes are increasingly being used as gene delivery and gene editing vectors for overcoming glioblastoma resistance mechanisms [88]. Engineered exosomes have been demonstrated to efficiently transport RNA-based therapeutics, including siRNA, miRNA, and CRISPR/Cas9 components, into brain tumor cells while preserving biological stability and minimizing immune clearance in vivo [116]. CRISPR-Cas9-loaded exosomes, in particular, have been investigated for silencing resistance-associated genes in glioblastoma, including pathways linked to MGMT-driven TMZ resistance and mesenchymal transition phenotypes in intracranial tumor models [122]. These systems highlight the adaptability of exosomes as biologically derived nanocarriers capable of crossing the BBB and delivering functional gene-editing machinery directly into brain tumor cells, thereby enabling precise molecular intervention in otherwise refractory glioma subtypes.

Surface modification strategies further expand the therapeutic versatility of exosomes by improving tumor tropism and enhancing penetration into the brain tumor microenvironment. Commonly used targeting moieties include neuropilin-1 (NRP-1) binding peptides [121], RGD motifs targeting integrins (αvβ3/αvβ5) [111], TAT peptides for enhanced cellular internalization [62], Angiopep-2 for BBB transport via LRP1 [62,117], and CD133 aptamers for glioma stem cell specificity [117]. These modifications collectively improve accumulation within orthotopic glioblastoma models, enhance intracellular uptake, and promote deeper penetration into invasive tumor margins, particularly in hypoxic and necrotic regions where conventional drug delivery is limited [62,117,121].

Importantly, a major advancement in this field is the integration of theragnostic exosome systems in glioblastoma, where the same engineered exosome platform is used both for therapy and for real-time tracking of biodistribution and tumor localization in the brain [118]. This aligns with the strict definition of theragnostic, in which a single molecular system simultaneously enables therapeutic delivery and diagnostic imaging of intracranial tumor targeting. Several imaging modalities have been successfully integrated into exosome platforms to achieve this goal in brain tumor models [118,123].

Fluorescent labeling strategies have been widely used for real-time in vivo tracking of exosome biodistribution in glioblastoma models. For example, fluorescent dye-labeled exosomes (e.g., DiR, Cy7) and genetically encoded fluorescent reporters (such as CD63-GFP fusion constructs) have been used to track exosome migration across the BBB and accumulation in orthotopic intracranial glioma sites, demonstrating clear tumor-selective enrichment compared with normal brain tissue [124,125]. To track these interactions, researchers developed a bio-orthogonal phospholipid labeling approach that enables fluorescent tagging of exosomes without altering membrane integrity or biological function. This platform allows dynamic imaging of organ distribution and tumor accumulation in live animal models, including tumor-bearing mice. This approach demonstrated preferential accumulation of systemically administered exosomes in tumor-bearing brain tissue, providing spatial and temporal resolution of biodistribution in vivo [125].

Bioluminescent imaging systems offer an additional layer of sensitivity for longitudinal tracking of exosomes in brain tumor models [126]. Exosomes engineered to carry luciferase-tagged membrane proteins (such as CD63-luciferase systems) enable non-invasive monitoring of vesicle distribution over time in living animals [127]. These systems allow repeated imaging without external excitation sources, reducing background autofluorescence and enabling quantitative assessment of exosome pharmacokinetics in deep brain tissues, including orthotopic glioblastoma xenografts [126,127].

Radiolabeling strategies have also been extensively applied for quantitative biodistribution analysis in glioblastoma models [128]. Exosomes labeled with positron-emitting isotopes such as copper-64 (^64^Cu) or zirconium-89 (^89^Zr) enable PET imaging to track systemic distribution, BBB crossing, tumor uptake, and clearance kinetics in real time [128,129]. Radiotracer labeling of small extracellular vesicles using ^89^Zr(oxinate)_4_ has enabled PET-based visualization of extracellular vesicle biodistribution and tumor localization with high sensitivity and quantitative accuracy in vivo [129]. Similarly, technetium-99m (^99^mTc)-labeled exosome-mimetic nanovesicles have been used in SPECT/CT imaging, enabling non-invasive visualization of biodistribution patterns and confirming vesicle transport into brain tissues and tumor-bearing regions [130]. These radionuclide-based systems are particularly valuable in neuro-oncology because they allow accurate quantification of vesicle passage across the BBB and retention within glioblastoma lesions [128,129].

More recently, magnetic resonance-based theragnostic systems have emerged for brain tumor imaging. Superparamagnetic iron oxide nanoparticles (SPIONs) were loaded into exosomes via electroporation, modified the exosome surface with a neuropilin-1-targeting peptide, and used the resulting platform for MRI-based tracking and imaging in orthotopic glioma models. These systems provide high spatial resolution and deep tissue imaging capability, making them particularly suitable for intracranial tumor models and brain metastatic disease [121].

Collectively, these studies demonstrate that engineered exosomes are evolving into fully integrated glioblastoma theragnostic systems capable of simultaneous targeted therapy delivery and non-invasive intracranial biodistribution imaging. In glioblastoma specifically, this dual functionality is critical for overcoming the major translation barrier of uncertain BBB delivery efficiency and heterogeneous tumor penetration. By combining ligand-mediated targeting (Angiopep-2, RGD, CD133 aptamers), gene-based therapy (RNA/CRISPR systems), and multimodal imaging (fluorescence, bioluminescence, PET/SPECT, MRI), exosome platforms provide a unified strategy for precision neuro-oncology that enables both therapeutic intervention and real-time validation of delivery efficiency within the brain tumor microenvironment.

##### Mechanistic and Translational Insights

As detailed in Section 5.1.1, tumor-derived exosomes play multifaceted roles in glioblastoma progression by transferring oncogenic cargo (e.g., EGFRvIII, miR-21) that drives proliferation, invasion, angiogenesis, metabolic reprogramming, and immune evasion—including M2 macrophage polarization and suppression of antitumor immunity. These same biological features—natural BBB crossing via transcytosis, cargo protection, and intrinsic tropism toward tumor cells—also make engineered exosomes highly attractive as therapeutic delivery vehicles [103,124,131]. However, the protumorigenic effects of unmodified tumor-derived exosomes highlight the importance of using engineered ‘factory’ cells (e.g., HEK293) or carefully modified vesicles to avoid unintended promotion of malignancy or immunosuppression.

For example, engineered exosomes targeting oncogenes such as MYC have been shown to reverse proneural-to-mesenchymal transition and extend survival in preclinical glioblastoma models [132]. In parallel, exosome-based vaccines derived from tumor cells have triggered strong antitumor immune responses in orthotopic brain tumor models, highlighting their potential beyond drug delivery [133].

At the same time, these advances bring important translational challenges into focus. Producing exosomes on a scale with consistent quality remains difficult because vesicle composition depends strongly on the originating glioma cell population and experimental conditions. Early glioblastoma studies demonstrated that tumor-derived extracellular vesicles carry heterogeneous RNA and protein cargo, including oncogenic EGFRvIII transcripts, which vary across tumor subclones and influence recipient cell behavior, highlighting intrinsic biological variability between preparations [101]. Such heterogeneity complicates standardization and limits reproducibility across laboratories and therapeutic platforms. In addition, vivo investigations show that systemically administered tumor-derived exosomes do not exclusively localize to the brain tumors but instead accumulate in peripheral organs such as the liver and spleen, indicating inefficient targeting despite their ability to cross the BBB [29,134]. Glioblastoma exosomes can also actively modulate non-tumor cells within the central nervous system; experimental studies demonstrate that they alter immune signaling and may induce neuronal stress responses, raising concerns about unintended biological effects beyond the tumor site [135]. Biodistribution control, off-target activity, and long-term safety therefore remain the most pressing unresolved questions standing between current preclinical progress and reliable clinical translation in neuro-oncology.

To address these issues, several engineering strategies are being developed within neuro-oncology research. Techniques such as large-scale cellular nanoporation have been applied to generate exosomes with enhanced RNA loading capacity and improved production yield, enabling efficient delivery of therapeutic mRNA to glioblastoma models while maintaining vesicle functionality [88]. To further overcome the inherent limitations of low production yields and restricted drug-loading capacity in native vesicles, hybrid exosome–liposome nanoparticles (HELNs) have been developed to merge exosomal biocompatibility with the high loading efficiency and tunable surface chemistry of synthetic liposomes [86]. A notable application of this strategy involves ‘pHybrid’ nanovesicles engineered through the fusion of blood-derived exosomes and tLyp-1 peptide-modified liposomes. These hybrids utilize transferrin receptor (TfR)-mediated transcytosis to penetrate the BBB and tLyp-1 peptides to target glioma cells, enabling the synergistic co-delivery of salvianolic acid B (SAB) and cryptotanshinone (CPT) to achieve enhanced anti-angiogenic and cytotoxic effects in the tumor microenvironment [87]. Membrane-fusion approaches have produced hybrid vesicles with increased drug encapsulation efficiency and enhanced antitumor activity in brain tumor models compared with native exosomes alone [136]. In parallel, advanced proteomic analyses of glioblastoma-derived extracellular vesicles are helping define cargo heterogeneity and identify functional protein signatures associated with tumor progression and intercellular signaling in the brain tumor microenvironment [101]. Complementing these approaches, bio-orthogonal labeling and imaging strategies now enable in vivo tracking of tumor-derived exosomes, allowing investigators to visualize biodistribution and quantify organ-specific accumulation following systemic administration in glioma-bearing animals [125]. These experimental advances are beginning to address key translation barriers by improving production scalability, mechanistic characterization, and real-time evaluation of exosome behavior in neuro-oncology settings.

Taken together, exosomes offer a flexible platform that connects mechanisms with application. They can function as both biomarkers and therapeutic carriers, with the ability to deliver drugs, nucleic acids, or immune signals in a targeted way in brain tumor models [137,138]. For example, exosome-mediated delivery of engineered microRNAs has been shown to suppress glioblastoma cell growth, reduce stem-like proliferation, and prolong survival in preclinical glioma models using exosomes engineered to carry therapeutic cargo [139]. Exosomes derived from neural stem cells have been used to transport antisense oligonucleotides targeting STAT3 into glioma microenvironments, enhancing immune responses and producing antitumor effects in mouse glioma models [140]. Similarly, Tao et al. demonstrated that exosomes derived from CAR-NK cells could serve as multifunctional “nano-bombs” for HER2-positive breast cancer brain metastases. These engineered exosomes were surface-functionalized with CAR molecules for selective tumor recognition and T7 peptides for BBB targeting, while encapsulating ROS-generating therapeutic nanoparticles. Systemic administration in mouse models resulted in efficient BBB crossing, preferential accumulation in brain metastases, strong tumor cell killing via ROS amplification and ferroptosis disruption, and significant survival benefits, all without observable systemic toxicity. This work illustrates how immune cell-derived exosomes can be adapted as targeted, brain-penetrant, multifunctional therapeutic platforms, complementing strategies developed in glioblastoma models [96]. While there are still hurdles to overcome, ongoing improvements in engineering and manufacturing are steadily moving exosome-based approaches closer to clinical use in glioblastoma and other brain tumors, with advanced strategies to cross the BBB and deliver bioactive cargo showing therapeutic efficacy in vivo [137,139]. Representative engineered exosome strategies for targeted therapy in glioblastoma are summarized in Table 2.

#### 5.1.2. The Role of Exosomes on Astrocytoma

Astrocytoma, a major subtype of gliomas, spans a spectrum from low-grade diffuse astrocytomas (often IDH-mutant) to anaplastic forms. In low-grade gliomas (which are dominated by astrocytic tumors), a specialized class of extracellular vesicles called spheresomes is produced at high levels. In human tumor specimens, spheresomes are more frequently found than classical exosomes, accumulate beneath the plasma membrane, and are released into the extracellular space, where they cross the BBB and may contribute to early tumor niche formation [141].

Tumor cells, including glioma and astrocytoma lineages, shed exosomes packed with bioactive molecules such as pro-invasive microRNAs (miRNAs), circular RNAs (circRNAs), proteins, and lipids into the surrounding microenvironment and circulation. These vesicles modulate recipient cells’ behavior and promote disease hallmarks such as angiogenesis, immune suppression, invasion, and tumor-niche establishment [141,142].

For example, exosomes isolated from glioma stem-like cells are enriched in regulatory miRNAs. In functional assays, exosomal miR-155-5p derived from glioma stem cells has been shown to drive mesenchymal transition and invasive behavior by targeting genes that control cell differentiation and migration [143]. Other exosomal miRNAs such as miR-26a promote angiogenesis through suppression of PTEN, activating pro-angiogenic PI3K/AKT signaling in endothelial cells within the tumor microenvironment [144]. These kinds of cross-cell regulatory mechanisms are highly relevant to astrocytic tumors because they shape cell–cell communication and support malignant progression.

Exosomal non-coding RNAs, including circRNAs, are selectively enriched in tumor-derived exosomes and alter gene regulatory networks in recipient cells. Deep sequencing studies of exosome RNA profiles from glioma microenvironment models reveal distinct signatures associated with aggressive phenotypes and poor prognosis, indicating that exosomal cargo reflects both tumor cell state and risk of malignant progression [92,142].

Because exosomes cross the BBB and contain tumor-derived molecular information, circulating exosomal miRNA and protein signatures have substantial value as non-invasive biomarkers for tumor grading, detection of malignant transformation, and prognostic stratification. In patients with low-grade gliomas, distinct exosomal miRNA profiles correlate with clinical outcomes and may provide insight into risk of progression to higher grade astrocytomas [92].

Engineered exosome strategies in astrocytoma overlap with those being developed for glioblastoma and other high-grade gliomas, because the challenges of BBB penetration and targeted delivery are shared across astrocytic tumors. Preclinical work demonstrates that exosomes can serve as delivery vehicles for tumor-suppressive miRNAs, chemotherapeutics, and gene modulation agents, achieving biological effects relevant to controlling tumor growth and therapy resistance [139].

Mesenchymal stem cell (MSC)-derived exosomes have been used experimentally to deliver tumor-suppressive miRNAs to glioma cells. In multiple glioma models, loading exosomes with antitumor miRNAs such as miR-124 reduces proliferation, alters oncogenic signaling, and enhances chemosensitivity. While this work is rooted in glioma biology, miR-124 delivery similarly targets pathways dysregulated in astrocytoma. miR-124 delivery inhibits proliferation and may reprogram glioma and astrocytoma cells toward less malignant states [89].

Exosomes can encapsulate chemotherapeutic agents and enhance drug delivery across the BBB. Preclinical studies show that exosomes loaded with small molecules penetrate the BBB more effectively than free drugs. In glioma xenograft models, exosome-mediated delivery of chemotherapeutics increases intratumoral drug accumulation and improves therapeutic responses relative to unencapsulated drugs. These findings suggest that exosome platforms could be adapted for delivering cytotoxic agents to astrocytoma cells while minimizing systemic toxicity [145].

Beyond direct tumor cell targeting, exosomes can alter the immune microenvironment. Tumor-derived exosomes can carry immunosuppressive miRNAs and proteins that downregulate antitumor immunity. Counteracting these effects, engineered exosomes with immune-stimulatory content are being explored to enhance antitumor immune responses. For example, two primary preclinical studies have demonstrated this potential in orthotopic glioma models through in vitro immune activation assays, detailed exosome engineering and characterization, and in vivo efficacy testing. The first study showed that neural stem cell (NSC)-derived exosomes engineered to carry CpG-STAT3ASO potently stimulate NF-κB signaling, IL-12 production, dendritic cell and macrophage activation, and antitumor immunity in the glioma setting. The second study utilized tumor cell-derived exosomes functionalized with CpG adjuvant (Exo-CpG) to reprogram the immunosuppressive tumor microenvironment, elicit innate and adaptive immune responses, inhibit GBM growth, and generate long-lasting protective immunity in multiple orthotopic models, including those of recurrence [97,140].

#### 5.1.3. Exosomes on Meningioma

Meningiomas represent the most prevalent primary intracranial tumors, primarily categorized as benign (WHO Grade 1). However, atypical (Grade 2) and malignant (Grade 3) variants present significant clinical challenges due to their increased recurrence rates and aggressive vascular behavior [146,147,148]. Emerging evidence suggests that the progression of these higher-grade variants is not solely determined by intrinsic mutations but is heavily influenced by the tumor microenvironment. Specifically, M2-polarized macrophages have been identified as key contributors; they secrete exosomes that function as critical intercellular messengers, driving tumor cell proliferation, migration, and invasion through the targeted activation of the TGF-β signaling pathway [149]. These extracellular vesicles are rich in bioactive cargo, including specific proteins such as talin-1, IQGAP1, fibrillin-1, and transgelin, which facilitate oncogenic remodeling, cytoskeletal reorganization, and angiogenesis within the niche [147].

Beyond their role in pathogenesis, exosomes and their encapsulated microRNAs are revolutionizing the diagnostic landscape as potent non-invasive biomarkers. For instance, the GATA-4/miR-497 axis serves as a molecular switch in high-grade meningiomas, where GATA-4-mediated downregulation of miR-497 correlates with increased malignancy; conversely, the presence of a validated 6-miRNA diagnostic panel in serum offers high sensitivity for distinguishing meningioma from other CNS pathologies [148,150]. Furthermore, the enrichment of plasma-derived EVs and the specific expression of markers such as Vimentin and CD147 provide a “liquid biopsy” signature that correlates with tumor volume and WHO grade [147]. This multifaceted role of exosomes underscores their potential both as therapeutic targets by disrupting M2 macrophage-to-tumor communication and as indispensable tools for the longitudinal monitoring of meningioma patients.

#### 5.1.4. Exosomes in Medulloblastoma

Medulloblastoma (MB) is an aggressive pediatric embryonal tumor characterized by four distinct molecular subgroups, WNT, SHH, Group 3, and Group 4, and a substantial potential for metastatic dissemination through cerebrospinal fluid (CSF) pathways [151,152]. Recent studies emphasize that the MB secretome is enriched with exosomes that facilitate critical intercellular communication within the tumor microenvironment (TME). These vesicles are loaded with bioactive cargo, including specific proteins such as B7-H3, GPNMB, and ERBB2, along with a variety of microRNAs that collectively drive tumor cell proliferation, migration, and the maintenance of cancer stemness. Furthermore, tumor-derived exosomes play a critical role in immune modulation by inducing M2-polarization in macrophages [105,153,154]. Conversely, exosomes derived from tumor-associated macrophages (TAMs) significantly influence immunotherapeutic sensitivity in the SHH-MB subgroup by modulating m6A-modified pathways; specifically, TAM-derived exosomal miRNAs downregulate the methyltransferase METTL14, leading to decreased global m6A levels and the subsequent upregulation of the transcription factor FOXD1, which suppresses cytotoxic T-cell recruitment [155].

The clinical utility of these vesicles is expanding into the realm of non-invasive diagnostics, as exosomal proteomic signatures found in serum and CSF hold significant promise for monitoring disease progression, metastasis, and recurrence [156,157]. Beyond diagnostics, engineered exosome platforms are gaining momentum as sophisticated therapeutic delivery vehicles. For instance, extracellular vesicles engineered with MB-targeting peptides and loaded with LOXL1-AS1 siRNA, especially when combined with focused ultrasound (FUS) to enhance BBB permeability, have demonstrated the ability to effectively suppress metastatic traits in SHH-subgroup models [158]. Additionally, CAR-NK or immune cell-derived exosomes targeting B7-H3 are emerging as alternative immunotherapeutic platforms, particularly for the aggressive Group 3 subtype [105,159]. Mesenchymal stem cell (MSC)-derived exosomes also provide a versatile delivery system for siRNA, miRNA, or other immunomodulatory cargoes [89,90]. These innovative strategies aim to complement standard multimodal therapies, potentially reducing metastatic dissemination while minimizing the long-term neurocognitive sequelae often associated with conventional pediatric treatments.

### 5.2. Stroke

Ischemic and hemorrhagic strokes cause rapid neural tissue damage accelerated by acute neuroinflammation, microvascular failure, and the accumulation of neurotoxic reactive oxygen species [160]. Preclinical studies show that engineered exosomes represent a powerful tool for post-stroke intervention due to their unique capacity to carry complex protective signals across the BBB [88]. Our laboratory has developed an engineering platform utilizing modified HEK293 cells that produce exosomes displaying the rabies virus glycoprotein (RVG) peptide on their surface while encapsulating neuroglobin payloads within their inner lumen [80]. When tested in animal models of middle cerebral artery occlusion, these targeted vesicles demonstrated robust homing to the ischemic penumbra, leading to a significant reduction in infarct volume and measurable improvements in long-term neurological scores compared to native, non-targeted control vehicles [80].

In addition to protein payloads, recent strategies focus on customizing the non-coding RNA profiles of exosomes to mitigate secondary ischemic injury. Enrichment of specific microRNAs within therapeutic vesicles has shown particular promise in this regard. Exosome-mediated transfer of miR-133b from mesenchymal stromal cells to neural cells has been shown to significantly increase neurite branch number and total neurite length in neurons exposed to post-stroke brain extracts, demonstrating that exosomal miRNA cargo can directly target downstream signaling cascades governing neuronal plasticity and recovery [91]. In parallel, M2 microglia-derived exosomes enriched with miR-124 attenuate ischemic brain injury and promote neuronal survival by suppressing the pro-apoptotic USP14 pathway, with knockdown of miR-124 in these exosomes substantially reversing their neuroprotective effects in a transient MCAO model [161]. Beyond direct neuronal effects, these engineered constructs actively reprogram the local microenvironment by shifting microglia from a pro-inflammatory M1 state toward a pro-resolving, neuroprotective M2 phenotype. By addressing both immediate oxidative damage and delayed neuroinflammatory cascades, engineered exosomes move beyond simple drug encapsulation to offer a multi-pronged therapeutic mechanism for neurovascular recovery. Exosomes derived from stem cells or endothelial progenitor cells (EPCs) have shown promise in mitigating ischemic injury by reducing inflammation, stabilizing the BBB, and promoting neovascularization [31,33,80].

### 5.3. Neurodegenerative Diseases

In neurodegenerative disorders such as Alzheimer’s and Parkinson’s diseases, engineered exosomes have been investigated as delivery vehicles for defined neuroprotective cargoes because they can cross the BBB and influence neuronal pathways. For instance, designer exosomes delivering catalase mRNA in Parkinson’s disease models attenuated oxidative stress, reduced neurotoxicity, and decreased neuroinflammation both in vitro and in vivo [73]. In Alzheimer’s disease models, engineered microglial exosomes enriched with miR-124-3p reduced Aβ plaque accumulation, dampened microglial and astrocyte activation, and improved cognitive outcomes by inhibiting the MEKK3/NF-κB pathway [162]. Similarly, exosomes loaded with miR-22 from adipose-derived mesenchymal stem cells enhanced neuronal survival, suppressed inflammatory factors, and improved memory and motor function in APP/PS1 mice [163], while miR-29b-2–containing exosomes targeted to hippocampal regions reduced presenilin-1 expression and β-amyloid accumulation [164]. Beyond their therapeutic potential, exosomal cargoes such as disease-associated miRNAs or proteins are being explored as biomarkers to monitor pathology and disease progression. While these preclinical results across Alzheimer’s, Parkinson’s, and stroke models are encouraging, they also underscore that glioblastoma remains the disease context where engineered exosome strategies are most mechanistically developed and translationally advanced. The disease-specific evidence reviewed in Section 5 points to several consistent biological properties that make exosomes particularly well suited for CNS delivery, and these are worth considering systematically before addressing the translational barriers.

### 5.4. Translational Progress: Clinical Trial Status of Exosome Therapeutics in CNS Disorders and Neuro-Oncology

To accurately map the current translational trajectory of extracellular vesicle therapies, it is vital to evaluate how preclinical successes are translating into active human clinical trials. While the vast majority of engineering strategies remain focused on optimization in rodent models, a growing number of phase 1 and phase 2 trials are establishing the human safety, tolerability, and early efficacy profiles of both natural and molecularly loaded exosome formulations [165,166]. These trials span across aggressive brain tumors like glioblastoma multiforme as well as severe acute or chronic neurodegenerative pathologies, moving the field beyond theoretical frameworks into the realm of viable clinical tools [166,167].

The clinical trials compiled in Table 3 reflect diverse strategies in donor-cell selection, delivery routes, and therapeutic cargo payloads. Notably, the field is leveraging both unengineered mesenchymal stem cell-derived exosomes, which capitalize on the innate anti-inflammatory and neuroprotective secretome of the parent cell [168,169], as well as heavily customized or engineered vesicles designed to shuttle target biomolecules [165,170].

Additionally, emerging approaches are exploring combinatorial techniques, such as utilizing transient transcranial focused ultrasound immediately prior to intravenous administration, to physically open localized regions of the BBB and enhance the target-specific homing of the circulating exosome therapeutic [170,171].

**Table 3:** Progress Report of Clinical Trials Utilizing Exosomes for CNS Disorders and Brain Tumors. The clinical trial data obtained from www.ClinicalTrials.gov (accessed on 1 June 2026).

Clinical Trial ID	Phase	Targeted Indication	Exosome Source	Therapeutic Cargo	Clinical Focus and Available Outcomes
NCT04202770 [172]	Not Applicable (Interventional, Open-Label)	Treatment-resistant depression, anxiety disorders, and neurodegenerative dementias	Allogeneic exosomes derived from healthy full-term cesarean-section amniotic fluid)	Native exosomal bioactive factors (growth factors, anti-inflammatory mediators); no specific engineered cargo reported	Designed to evaluate the safety, feasibility, and potential efficacy of intravenous exosome administration combined with transcranial focused ultrasound to enhance targeted delivery to the brain. Ultrasound targeting was directed to the subgenual cingulate (depression), amygdala (anxiety), or hippocampus (dementia). Primary outcomes included changes in Beck Depression Inventory (BDI-II), Beck Anxiety Inventory (BAI), Quick Dementia Rating Scale (QDRS), and Global Rating of Change (GRC) scores at 8 weeks. The trial was subsequently listed as suspended, and no peer-reviewed efficacy or final clinical outcome data have been reported on ClinicalTrials.gov.
NCT04573140 [173]	Phase I	Newly diagnosed adult glioblastoma (GBM) and pediatric high-grade glioma (pHGG)	Not applicable (RNA-loaded lipid particle vaccine; not exosome-based)	Autologous total tumor mRNA plus pp65 full-length lysosomal-associated membrane protein (LAMP) mRNA encapsulated in DOTAP lipid particles	First-in-human study designed to evaluate manufacturing feasibility, safety, and maximum tolerated dose (MTD) of personalized RNA-LP vaccines. The primary endpoints include successful vaccine manufacture, safety assessment, and dose escalation. As of the latest registry update, efficacy results and clinical outcomes have not yet been reported because the trial remains ongoing/recruiting.
NCT05559177 [174]	Early Phase I (open-label, dose-escalation)	Recurrent or metastatic bladder cancer refractory to conventional therapies	Personalized chimeric exosomal tumor vaccines generated from patient-derived bladder tumor cells fused with autologous antigen-presenting cells (dendritic cells or macrophages)	Native tumor antigens incorporated within chimeric exosomes designed to stimulate antitumor immune responses	First-in-human study evaluating the safety, tolerability, dose-limiting toxicity (DLT), maximum tolerated dose (MTD), and preliminary antitumor activity of chimeric exosomal tumor vaccines. Primary endpoints include clinical response rate, overall survival, and treatment-related adverse events. The study also aims to characterize immune activation and modulation of the tumor microenvironment. As of the latest ClinicalTrials.gov update, no efficacy or outcome results have been posted.
NCT03608631 [98]	Phase I (with planned Phase II expansion)	Metastatic pancreatic ductal adenocarcinoma (PDAC) harboring the KRAS^G12D^ mutation	Mesenchymal stromal cell (MSC)-derived exosomes (iExosomes)	KRAS^G12D^-targeting siRNA loaded into MSC-derived exosomes	First-in-human clinical trial evaluating the safety, tolerability, dose-limiting toxicities (DLTs), maximum tolerated dose (MTD), pharmacodynamics, and preliminary antitumor activity of intravenously administered KRAS^G12D^ siRNA-loaded exosomes. Secondary endpoints include progression-free survival (PFS), overall survival (OS), target engagement, and assessment of KRAS pathway inhibition. The study remains active, and no peer-reviewed efficacy results or final clinical outcomes have yet been reported.
NCT05354141 [175]	Phase III	Moderate-to-severe Acute Respiratory Distress Syndrome (ARDS)	Bone marrow mesenchymal stem cell (MSC)-derived extracellular vesicles (ExoFlo^®^)	Native MSC-derived extracellular vesicle cargo containing endogenous regenerative, immunomodulatory, and anti-inflammatory bioactive molecules	Multicenter, randomized, double-blind, placebo-controlled trial evaluating the safety and efficacy of intravenous ExoFlo^®^ in hospitalized patients with moderate-to-severe ARDS. The primary endpoint is 60-day all-cause mortality. Secondary endpoints include time to death, ventilator-free days, oxygen-free days, ICU-free days, and treatment-emergent serious adverse events. As of the latest ClinicalTrials.gov update (February 2026), the study remains active/recruiting, and no final efficacy results have been publicly reported.

Analyzing this active pipeline reveals three significant insights regarding the translation of neuro-oncology exosome platforms. First, safety serves as the primary milestone, as early-phase data consistently show that allogeneic and autologous exosome formulations lack severe systemic infusion toxicities, displaying a superior safety profile compared to intact living stem cell therapies [176,177]. Second, the field is seeing a rise in hybrid delivery; trials like NCT04202770 demonstrate that chemical or physical disruption methods, such as focused ultrasound, are increasingly coupled with exosome delivery to maximize brain parenchyma accumulation and bypass natural hepatic or splenic clearance [178,179]. Third, there is a strong regulatory focus on source characterization, meaning the primary bottleneck highlighted across these ongoing trials is not clinical toxicity, but rather the strict requirement for precise characterization of donor cells, which dictates the consistency of the final therapeutic batch [177,180].

## 6. Advantages of Exosomes as a Targeted Drug Delivery System

Exosomes possess several properties that distinguish them from conventional drug delivery platforms and support their development as therapeutic carriers for CNS disorders and neuro-oncological applications. Unlike synthetic nanoparticles, exosomes are naturally occurring extracellular vesicles that have evolved to mediate intercellular communication, enabling efficient interactions with biological systems while maintaining high biocompatibility and relatively low immunogenicity. Their endogenous origin reduces recognition by the mononuclear phagocyte system and contributes to prolonged circulation times in vivo. Furthermore, membrane-associated proteins such as CD47 can promote immune evasion by transmitting “don’t-eat-me” signals to phagocytic cells, thereby reducing premature clearance and enhancing systemic persistence [181].

A major advantage of exosomes is their ability to overcome biological barriers that frequently limit the effectiveness of conventional therapeutics. As discussed in Section 2.2, exosomes can traverse the blood–brain barrier through endogenous transport mechanisms, including receptor-mediated uptake and transcytosis. This intrinsic capability enables more effective delivery of therapeutic cargo to CNS tissues than many synthetic nanocarriers, which often require extensive surface modification to achieve comparable levels of brain accumulation [64,73,182]. In addition, the phospholipid bilayer of exosomes protects encapsulated proteins, nucleic acids, and other sensitive biomolecules from enzymatic degradation during circulation, thereby improving cargo stability and bioavailability.

The therapeutic versatility of exosomes is further enhanced by their capacity for both intrinsic and engineered targeting. Natural tissue tropism may facilitate selective accumulation in specific biological environments, while surface-engineering approaches can further improve targeting precision. Strategies such as peptide display, receptor engineering, aptamer conjugation, and bio-orthogonal surface modification have enabled enhanced delivery to disease-relevant cell populations while minimizing off-target exposure. These capabilities have supported the development of exosome-based platforms for the delivery of chemotherapeutics, RNA therapeutics, proteins, and genome-editing systems across a broad range of CNS disease models [64,183].

Despite these advantages, important challenges remain before exosome therapeutics can achieve widespread clinical implementation. In contrast to synthetic nanocarriers, which benefit from well-established manufacturing pipelines and precise control over drug loading and release kinetics, exosome-based systems continue to face obstacles related to scalable production, batch-to-batch variability, purification, characterization, and standardization [184,185,186]. Furthermore, donor-cell selection, cargo heterogeneity, and biodistribution variability may influence therapeutic consistency and regulatory approval.

Taken together, engineered exosomes offer a unique combination of biological compatibility, immune tolerance, cargo protection, BBB penetration, and targeting flexibility that is difficult to replicate using conventional delivery systems. While substantial translational challenges remain, their ability to integrate natural biological functionality with advanced engineering strategies positions exosomes as one of the most promising platforms for precision drug delivery in neuro-oncology and other CNS disorders [64,73,181,182,183,184,185,186].

## 7. Barriers to Clinical Translation and Emerging Strategies

Despite the significant biological advantages of engineered exosomes described in previous sections, multiple translational barriers continue to limit their widespread clinical implementation. These challenges include scalable manufacturing, purification consistency, cargo loading efficiency, targeting specificity, biological heterogeneity, immunogenicity, and incomplete regulatory standardization. Importantly, these limitations are highly interconnected, such that improvements in one area frequently influence performance in others. Consequently, successful clinical translation will require integrated strategies that simultaneously address manufacturing, engineering, safety, and regulatory considerations.

Low production yield remains one of the primary technical bottlenecks preventing the clinical translation of extracellular vesicle platforms from benchtop concepts to industrial-scale manufacturing [16,85,99,100,187,188,189]. While baseline biology establishes that the fate of a multivesicular body (MVB) is determined by a competitive molecular tug-of-war between Rab27-mediated plasma membrane secretion and Rab7-dependent lysosomal degradation, modern precision medicine requires engineering strategies designed to intentionally break this intracellular balance [51,56,61]. By systematically overexpressing specific small GTPases or applying targeted exogenous stimuli, investigators can actively tilt the cellular scales to favor the secretory pathway over degradative pathways.

Genetic modification of donor factory cells represents the most direct method to achieve substantial yield enhancements. Overexpression of the small GTPases Rab27a and Rab27b accelerates the critical trafficking and docking steps of mature secretory MVBs at the cell periphery [56]. This genetic amplification ensures that a higher percentage of intracellular vesicles are pushed toward exocytosis before they can be intercepted by degradative lysosomal machinery. Furthermore, engineering donor cells to overexpress Rab31 provides an alternative, Endosomal Sorting Complex Required for Transport (ESCRT)-independent route that significantly increases vesicle output [52]. Rab31 functions as a dual-action switch because it simultaneously drives intraluminal vesicle budding along lipid rafts and actively suppresses MVB fusion with lysosomes, thereby shunting a vast majority of the endosomal pool toward immediate extracellular release [52,55].

Complementing these permanent genetic alterations, the utilization of external chemical and biological stimuli provides an alternative, highly adjustable layer of control over the cellular secretory apparatus. Exogenous stimuli that induce transient intracellular calcium elevations can be strategically deployed during bioreactor processing to maximize harvest concentrations. For instance, calcium influx activates Munc13-4, an essential calcium-binding effector that physically interacts with Rab27 complexes [57]. This calcium-induced activation accelerates assembly with core soluble N-ethylmaleimide-sensitive factor attachment protein receptor (SNARE) complexes, including syntaxin-4, SNAP-23, and VAMP-7, which greatly minimizes the docking time required for vesicle fusion at the plasma membrane [57,58]. Integrating these specific engineering approaches into active production protocols moves the field away from purely observational biology, establishing a mechanistic framework that turns donor cells into hyper-secretory therapeutic factories capable of sustaining human clinical pipelines.

### 7.1. Production, Scalability, and Standardization

One of the major barriers to exosome therapeutics is the absence of scalable and reproducible manufacturing systems capable of generating clinically relevant quantities of highly purified vesicles. Conventional isolation approaches, including differential ultracentrifugation and precipitation-based methods, often produce heterogeneous extracellular vesicle populations with variable purity, low recovery, and limited scalability. These limitations contribute to substantial batch-to-batch variability and complicate quality control, regulatory evaluation, and therapeutic reproducibility [17,20].

To overcome these challenges, increasing attention has focused on scalable bioprocessing strategies, including hollow-fiber bioreactors, stirred-tank systems, and three-dimensional culture platforms capable of enhancing extracellular vesicle yield while maintaining vesicle integrity. Bioreactor-based culture systems have demonstrated improved production efficiency and may facilitate transition toward GMP-compatible manufacturing pipelines [20,189].

Purification and downstream processing remain equally important challenges for clinical translation. Although ultracentrifugation remains widely used in laboratory-scale studies, it is associated with limited scalability, vesicle aggregation, and inconsistent recovery. In contrast, tangential flow filtration (TFF) enables high-throughput concentration and purification of extracellular vesicles from large sample volumes while preserving vesicle structure and biological activity [99,100]. Similarly, size-exclusion chromatography (SEC) has emerged as an effective method for improving vesicle purity and reducing contamination by soluble proteins and non-vesicular extracellular particles [99]. Increasingly, combined TFF-SEC workflows are being implemented to improve both scalability and reproducibility in translational applications.

#### 7.1.1. Comparative Analysis of Industrial Purification Protocols and Scalability Standards

While traditional laboratory-scale workflows involving 0.22 µm filtration followed by 50 to 100 kDa molecular weight cut-off centrifugal filtration, offer rapid and reliable processing for preclinical exploratory studies, translating exosome therapies to clinical-grade production demands a transition toward automated, highly reproducible industry-standard purification technologies [99,100,187,188,189]. Achieving regulatory approval requires rigorous compliance with safety and purity criteria, making it essential to thoroughly evaluate how different isolation platforms impact vesicle structural integrity, downstream cargo stability, overall processing yield, and batch-to-batch consistency [16,44,85,187,188].

#### 7.1.2. Ultracentrifugation (UC)

Ultracentrifugation remains the historical baseline and most widely implemented method for isolating extracellular vesicles, relying on sequential hydrodynamic separation based on particle density and size [44,99,100,187]. While differential ultracentrifugation is effective for processing small-volume laboratory samples, it presents significant limitations for scale-up operations and clinical translation [44,100,187]. The primary disadvantage of this technique is the generation of extreme, prolonged shear forces that can physically disrupt the exosomal lipid bilayer, leading to vesicle rupture, leakage of therapeutic nucleic acid or protein cargo, and the formation of uncharacteristic vesicular aggregates [44,187]. Furthermore, ultracentrifugation frequently co-isolates non-vesicular macromolecular contaminants, such as high-density lipoproteins and abundant plasma proteins like albumin, which compromises the purity of the final therapeutic lot [44,187]. From a manufacturing perspective, the process is highly labor-intensive, lacks automated inline monitoring, and exhibits severe batch-to-batch variability driven by minor inconsistencies in rotor deceleration profiles and operator handling [44,100,187,188,189].

#### 7.1.3. Tangential Flow Filtration (TFF)

Tangential flow filtration has emerged as a preferred industrial standard for the rapid concentration and initial clarification of large-volume conditioned media [99,100,188,189]. Unlike dead-end filtration systems, where liquid flows perpendicular to the membrane surface and causes rapid filter clogging, TFF directs the fluid stream parallel to the filter membrane [99,100]. This tangential flow creates a continuous sweeping motion that minimizes particle accumulation, reduces cake formation, and allows for gentle, high-throughput processing [99,100,187]. TFF is exceptionally efficient at removing bulk water, low-molecular-weight proteins, and media supplements while preserving the native structural integrity of the delicate exosome membrane [44,99,100,187]. Because the fluid dynamics can be tightly controlled via automated pump systems, TFF significantly reduces the mechanical stress exerted on the vesicles, thereby preserving the structural stability of both surface-engineered homing peptides and internally encapsulated molecular payloads [44,85,187]. The scalability of TFF makes it an indispensable component of upstream clinical manufacturing, although it is typically coupled.

#### 7.1.4. Size-Exclusion Chromatography (SEC)

Size-exclusion chromatography represents the gold standard for high-purity isolation, separating components based purely on their hydrodynamic radius as they pass through a porous stationary phase matrix [44,99,187,188]. Large macromolecular vesicles travel rapidly around the porous beads and elute early in the void volume, while smaller soluble proteins and free nucleic acids enter the matrix pores and elute in later fractions [44,187]. This gravity or low-pressure separation completely avoids the damaging shear stresses inherent to ultracentrifugation, ensuring that the isolated exosomes maintain their authentic biological morphology, surface charge, and cargo functionality [44,187]. The primary advantage of SEC is its unmatched ability to cleanly separate intact exosomes from free-floating protein complexes, delivering a highly purified product with exceptional batch-to-batch consistency [44,187]. However, when utilized as a standalone method, SEC suffers from low throughput, potential sample dilution during elution, and a limited capacity to handle large raw input volumes, which can bottleneck continuous industrial bioprocessing [99,100,187,188].

A summary of these exosome purification strategies is given in Table 4.

To satisfy the stringent purity and reproducibility mandates of regulatory agencies for clinical-grade therapeutics, current manufacturing trends favor a hybrid, multidimensional purification pipeline [99,100,187,188]. The optimal industrial recommendation involves utilizing tangential flow filtration as an initial upstream step to achieve rapid volume reduction and bulk concentration, immediately followed by downstream size-exclusion chromatography as a precision polishing step to eliminate residual non-vesicular proteins and achieve maximum purity [99,100,187]. This integrated, closed-loop approach successfully mitigates batch-to-batch variability, stabilizes the therapeutic cargo, and ensures the continuous, scalable production of uniform exosome batches suitable for human clinical evaluation [99,100,187,188,189].

Several groups, including our own, have developed scalable workflows integrating membrane filtration, ultrafiltration, centrifugal concentration, and donor-cell engineering approaches to generate therapeutic exosomes with enhanced targeting capabilities. For example, engineered HEK293-derived exosomes displaying RVG peptides have demonstrated improved BBB targeting and CNS delivery in preclinical models. However, despite these advances, fully standardized GMP-compliant manufacturing pipelines remain incompletely established, and harmonized quality-control criteria are still lacking across the field.

In response to these challenges, the International Society for Extracellular Vesicles (ISEV) recently updated the MISEV2023 guidelines, which provide expanded recommendations for extracellular vesicle production, separation, characterization, storage, and functional validation [187]. Adoption of standardized manufacturing and characterization frameworks is expected to improve reproducibility, facilitate inter-study comparison, and accelerate regulatory acceptance of exosome-based therapeutics.

### 7.2. Cargo Loading Efficiency and Stability

Efficient and stable cargo loading remains a central engineering challenge for therapeutic exosomes. Physical loading approaches such as electroporation, sonication, extrusion, and freeze–thaw cycling can facilitate incorporation of therapeutic molecules but may simultaneously disrupt vesicle membranes, alter cargo composition, or induce aggregation of nucleic acids. Electroporation-based loading of siRNA and miRNA, for example, has been associated with RNA aggregation artifacts that may lead to overestimation of encapsulation efficiency [65,73].

Conversely, gentler passive loading approaches better preserve vesicle integrity but frequently achieve insufficient loading efficiency for therapeutic applications. This reflects a persistent trade-off between cargo incorporation efficiency and preservation of vesicle structure and biological activity [73,190].

Recent advances in endogenous and genetically programmable loading systems have improved cargo specificity while minimizing structural disruption. Examples include EXPLOR-based protein loading systems, CD63- and Lamp2b-mediated cargo recruitment approaches, and RNA-binding protein-guided packaging strategies that enable selective incorporation of therapeutic proteins and nucleic acids into extracellular vesicles [66,71]. These programmable systems offer improved reproducibility and may enhance the translational feasibility of engineered exosome therapeutics.

### 7.3. Targeting Specificity and Biodistribution

Although substantial progress has been made in exosome surface engineering, achieving precise in vivo targeting remains a major limitation. Following systemic administration, a large proportion of extracellular vesicles accumulate in off-target organs, particularly the liver, spleen, and lungs, thereby reducing therapeutic delivery to intended tissues [134].

To improve targeting specificity, multiple engineering strategies have been developed, including peptide display, receptor-mediated targeting, aptamer conjugation, and bio-orthogonal surface modification. Neuronal targeting peptides such as rabies virus glycoprotein (RVG) have demonstrated enhanced BBB penetration and neuronal uptake in preclinical CNS models, whereas integrin-binding peptides such as RGD and transferrin receptor-targeting approaches have improved tumor and endothelial targeting [64,79,183]. Additional studies have explored CD206-targeted exosomes for selective delivery to tumor-associated macrophages and inflammatory myeloid populations within the tumor microenvironment.

More recently, multivalent targeting strategies incorporating multiple ligands on a single exosome surface have emerged as a promising approach for improving tissue specificity and cellular uptake [62]. Computational modeling and machine learning-based prediction of biodistribution patterns are also being investigated to optimize targeting efficiency and minimize off-target accumulation [191]. Nevertheless, despite encouraging preclinical data, reproducible and tissue-specific delivery following systemic administration remains incompletely controlled and continues to represent a major translational challenge.

### 7.4. Biological Variability, Safety, and Immunogenicity

Exosomes exhibit substantial biological variability depending on their cellular origin, physiological state, and environmental conditions. This heterogeneity contributes to inconsistent functional properties across preparations and complicates standardization. In addition, their complex molecular cargo introduces potential risks of unintended biological activity or immune activation, particularly when derived from poorly characterized donor cells. These considerations underscore the importance of rigorous characterization and standardized handling procedures when developing exosome-based therapeutics.

The absence of standardized protocols for isolation, characterization, and storage further exacerbates variability and limits reproducibility. To address this issue, the International Society for Extracellular Vesicles has established community guidelines through the Minimal Information for Studies of Extracellular Vesicles (MISEV2018) framework, which recommends transparent reporting of isolation methods, multiparametric characterization of vesicle size and composition, validation using positive and negative protein markers, and functional assays confirming vesicle-specific biological activity [44]. Adoption of these standardized criteria has improved experimental rigor across the field and provides an emerging foundation for translational and regulatory consistency. Alignment with such guidelines is increasingly recognized as essential for ensuring reproducibility and enabling comparison across studies.

Consequently, comprehensive preclinical safety assessment remains essential, particularly in the context of repeated systemic administration, to better define immunological and long-term safety profiles. Notably, accumulating experimental evidence indicates that properly characterized exosomes can exhibit low immunogenicity in vivo. Reports demonstrate that long-term administration of HEK293-derived engineered exosomes does not increase immunogenic responses, supporting their safety under repeated dosing conditions [127]. These observations are consistent with multiple primary studies reporting minimal immune activation, absence of significant inflammatory cytokine induction, and lack of organ toxicity following systemic delivery of purified HEK293 cell-derived extracellular vesicles [127,188]. Together, these data suggest that immunogenicity is not an inherent limitation of exosome therapeutics but instead depends strongly on donor-cell selection, purification quality, and adherence to standardized characterization practices.

At the same time, improved understanding of exosome–immune system interactions and the development of immunologically “stealth” or autologous exosome platforms represent promising approaches to further reduce immunogenic risk and improve therapeutic safety [181]. Continued integration of standardized experimental frameworks with mechanistic studies of immune compatibility will be critical for minimizing biological variability and strengthening the clinical translation of engineered exosome therapies.

### 7.5. Regulatory Challenges

Regulatory frameworks for exosome-based therapeutics remain incompletely defined and represent a major non-biological barrier to clinical translation. Key unresolved issues include product classification (biologic versus drug delivery system), absence of standardized potency and characterization assays, and limited regulatory consensus regarding pharmacokinetics, biodistribution, and dosing strategies. Like other biologically derived therapeutics, exosomes are heterogeneous products whose composition depends on donor-cell identity, culture conditions, and manufacturing workflows, complicating conventional regulatory evaluation based solely on molecular definition.

These challenges parallel those encountered during the early clinical development of stem cell-based therapies, where regulators faced comparable uncertainty regarding product identity, mechanism of action, and batch variability. Regulatory progress achieved for mesenchymal stromal cell (MSC) therapies provides a practical framework for reducing the translational burden for engineered exosomes. For example, approval of allogeneic MSC products such as remestemcel-L for pediatric graft-versus-host disease and darvadstrocel (Alofisel) for treatment-refractory Crohn’s disease-associated fistulas demonstrated that complex biological therapies can be evaluated using risk-based regulatory models emphasizing manufacturing consistency, safety, and functional potency rather than complete molecular characterization alone [192,193]. These approvals established precedents in which validated production processes and reproducible biological activity served as primary regulatory anchors rather than precise compositional uniformity.

Applying a similar strategy to exosome therapeutics would shift emphasis toward standardized manufacturing pipelines and validated release criteria. Alignment with internationally accepted extracellular vesicle standards, particularly the Minimal Information for Studies of Extracellular Vesicles (MISEV) guidelines, can provide a foundation for regulatory harmonization by defining minimal characterization parameters such as vesicle size distribution, enrichment of canonical markers, cargo consistency, sterility testing, and functional bioactivity assays [44]. This process-oriented approach mirrors quality control frameworks successfully implemented in cell therapy manufacturing and directly addresses the variability concerns discussed in Section 7.4.

Regulatory experience from advanced therapy medicinal products (ATMPs) further suggests that staged clinical development pathways may reduce translational barriers. Stem cell therapies frequently advanced through adaptive regulatory models, allowing early-phase clinical testing under controlled manufacturing conditions while analytical standards continued to mature. A comparable phased pathway for engineered exosomes, beginning with safety- and biodistribution-focused trials followed by iterative refinement of potency assays and manufacturing controls, could accelerate clinical entry while maintaining regulatory rigor [188].

In addition, early and continuous interaction between academic investigators, industry partners, and regulatory agencies, a strategy widely adopted during MSC therapy development, may help define acceptable endpoints for potency, biodistribution, and dosing before late-stage trials. Such proactive regulatory engagement reduces uncertainty, minimizes costly redevelopment of manufacturing workflows, and promotes convergence toward harmonized expectations across jurisdictions [194].

Taken together, lessons learned from stem cell regulatory approvals indicate that exosome therapeutics need not await complete biological standardization before clinical translation. Instead, adoption of risk-based evaluation, standardized characterization frameworks, and adaptive regulatory pathways can reduce current barriers. Establishing harmonized guidelines for manufacturing, quality control, and functional characterization, informed by successful precedents in cell therapy regulation, will be essential for enabling efficient and predictable clinical advancement of engineered exosome-based therapeutics.

### 7.6. Integrated Emerging Strategies and Future Directions

Emerging solutions in the exosome field increasingly focus on integrated engineering strategies capable of simultaneously addressing manufacturing, targeting, cargo loading, and safety limitations. Rather than functioning as isolated technological advances, these approaches collectively aim to improve translational feasibility and therapeutic reproducibility.

One major direction involves the development of scalable and standardized manufacturing platforms integrating bioreactor-based culture systems with GMP-compatible downstream purification approaches such as tangential flow filtration and size-exclusion chromatography [20,99,100]. These systems are expected to improve production consistency, reduce heterogeneity, and facilitate large-scale clinical manufacturing.

In parallel, programmable cargo loading technologies and modular surface-engineering strategies are enhancing therapeutic precision. Endogenous RNA packaging systems, engineered RNA-binding proteins, EXPLOR-based protein loading methods, and bio-orthogonal membrane engineering approaches are improving cargo specificity while preserving vesicle stability and biological functionality [66,71]. Such advances may substantially improve delivery efficiency and reduce off-target effects in future therapeutic applications.

A growing understanding of exosome-immune system interactions is also guiding the development of immunologically optimized vesicles. Approaches utilizing autologous donor cells, engineered “stealth” exosomes, and immune-evasive membrane proteins may reduce clearance by the mononuclear phagocyte system and improve long-term safety profiles [181].

Finally, personalized and theragnostic exosome systems represent an important future direction for precision neuro-oncology. Personalized exosomes carrying patient-specific RNA or protein payloads may enable individualized treatment strategies for heterogeneous diseases such as glioblastoma, while theragnostic exosomes combining therapeutic and imaging capabilities may facilitate real-time monitoring of biodistribution and treatment response. Integration with advanced imaging technologies is expected to further enhance the translational utility of engineered exosome platforms [119,120].

These interconnected challenges and corresponding solution strategies are synthesized in Table 5.

## 8. Conclusions

Engineered exosomes have demonstrated clear biological advantages for CNS drug delivery, but translating that promise into clinical practice will require resolving persistent challenges in scalable production, cargo loading efficiency, targeting specificity, safety, and regulatory standardization, as outlined in Section 7.

The most important near-term priorities are improving manufacturing consistency and developing validated quality metrics that can be applied across different cell sources and engineering platforms. Without reproducible production, the mechanistic advances reviewed here (in biogenesis control, endogenous cargo loading, surface targeting, and theragnostic integration) cannot be reliably transferred into clinical-grade material. Standardization is therefore not just a manufacturing problem but a prerequisite for meaningful clinical trial design.

Looking further ahead, the convergence of programmable cargo loading, AI-guided surface engineering, and patient-specific exosome design offers a realistic path toward truly personalized CNS therapeutics. The work reviewed here suggests that engineered exosomes are no longer simply an interesting biological concept. The mechanistic foundation is solid enough that the remaining barriers are primarily engineering and regulatory problems, which are, in principle, solvable. Continued interdisciplinary collaboration between cell biologists, nanotechnologists, clinicians, and regulatory scientists will be essential to close that gap.

## Figures and Tables

**Table 1:** Engineering strategies for exosome cargo loading and targeting.

Strategy Category	Method	Mechanism	Suitable Cargo	Key Advantages	Limitations	Representative Examples
Endogenous loading	Genetic engineering (EXPLOR, EXOtic)	Cargo incorporated during exosome biogenesis via protein or RNA sorting pathways	Proteins, mRNA	High specificity, stable incorporation	Requires donor-cell modification, lower yield	[66,71]
Exogenous loading	Electroporation	Temporary membrane permeabilization allows cargo entry	siRNA, miRNA	Simple, widely used	RNA aggregation, variable efficiency	[65]
	Sonication	Mechanical disruption enhances membrane permeability	Small molecules, proteins	High loading efficiency	May affect vesicle integrity	[72]
	Extrusion	Vesicle deformation and reassembly with cargo	Proteins, drugs	Uniform size distribution	Structural alteration risk	[73]
	Lipid-based transfection	Fusion of lipid–cargo complexes with exosome membrane	RNA, DNA nanostructures	Preserves vesicle structure	Lower loading efficiency	[74]
Surface functionalization	Genetic fusion	Targeting ligand displayed on exosome membrane proteins	Peptides	Specific targeting, biologically integrated	Limited control of ligand density	Lamp2b (exosomal membrane protein) + RVG peptide [64] Signal peptide + CD206-targeting peptide + Fc portion of mouse IgG2b + PDGFR transmembrane domain [79]
	Click chemistry	Covalent attachment of ligands to membrane components	Peptides, aptamers	Precise and versatile modification	Stability and scalability concerns	[75,78]
	Antibody/aptamer conjugation	Receptor-specific binding to target cells	Antibodies, aptamers	High targeting specificity	Potential immunogenicity	[82,83]

**Table 2:** Representative engineered exosome-based strategies for targeted therapy in glioblastoma.

Strategy/Study	Cargo Type	Targeting Mechanism	Model	Key Therapeutic Outcome
Cetuximab + Doxorubicin Exosomes [110]	Chemotherapeutics (cetuximab, doxorubicin)	Tumor targeting via intrinsic exosome tropism	Glioma-bearing rats	Enhanced BBB penetration, improved tumor cytotoxicity, prolonged survival
RGD-Modified Exosomes [62,111]	Small molecules/siRNA	RGD peptide fused to exosome membrane proteins	Orthotopic GBM mouse model	Increased tumor accumulation and reduced off-target toxicity
Glioblastoma-Derived Exosomes carrying Selumetinib [113]	Chemotherapy (selumetinib)	Innate tumor tropism	In vivo GBM xenograft	Efficient targeting without external ligands and improved therapeutic efficacy
Aptamer-Functionalized Exosomes [117]	Temozolomide + O^6^-benzylguanine	Angiopep-2 peptide and CD133 RNA aptamer	U87MG cells and GSC mouse model	BBB penetration, glioma stem cell targeting, reversal of drug resistance
CRISPR-Cas/RNA Delivery Exosomes [122]	CRISPR-Cas9, siRNA, TMZ, inhibitors	Engineered exosome targeting system	Patient-derived GBM cells and mouse models	Gene modulation and reversal of TMZ resistance in mesenchymal GBM
Theragnostic Exosomes [119,120]	Chemotherapy + imaging labels	Surface ligand functionalization	Preclinical GBM models	Simultaneous tumor imaging and therapy monitoring
T7 Peptide-Modified Exosomes [116]	AMO-21 (anti-miR-21 oligonucleotide)	T7 peptide targeting transferrin receptor	GBM cell lines and orthotopic mouse model	miR-21 inhibition and reduced tumor growth
Iron-Oxide Labeled Exosomes [110]	Imaging nanoparticles ± drugs	Magnetic labeling enabling tracking	Preclinical GBM models	Real-time biodistribution tracking using magnetic particle imaging
Exosome–Liposome Hybrid Nanovesicles [86]	Chemotherapeutics/nucleic acids	Hybrid membrane engineering	Orthotopic GBM models	Improved stability, drug loading efficiency, and tumor delivery
CAR-NK Cell-Derived Exosome “Nano-bombs” [96]	Cytotoxic proteins and immune modulators	Immune cell-derived exosome targeting	GBM mouse models	Enhanced immune-mediated tumor killing with reduced systemic toxicity
Cellular Nanoporation mRNA-Loaded Exosomes [88]	Therapeutic mRNA	High efficiency nanoporation loading	In vitro and in vivo GBM models	Increased protein expression and therapeutic delivery efficiency
Ferroptosis-Inducing Exosome Conjugates [115]	Ferroptosis-triggering agents	Surface-engineered targeting ligands	GBM preclinical models	Induced lipid peroxidation and tumor cell death
NIR-II Imaging Theragnostic Exosomes [88]	Chemotherapy + NIR-II fluorophores	Optical imaging-guided targeting	Orthotopic GBM models	Deep tissue imaging with precise therapy monitoring
siRNA-Loaded Targeted Exosomes [108]	Gene-silencing RNA	Ligand-mediated tumor targeting	GBM xenograft models	Downregulation of oncogenic pathways and reduced tumor growth
Stem-Cell Tropic Engineered Exosomes [107]	TMZ + resistance-modulating nucleic acids	Stem cell marker targeting ligands	Glioblastoma stem cell models	Overcame chemoresistance and improved therapeutic response
Fluorescent Theragnostic Exosome Platforms [114]	Drugs + fluorescent reporters	Surface functionalization	Preclinical GBM models	Concurrent treatment and visualization of treatment response

**Table 4:** Summary Recommendation for Clinical-Grade Production.

Purification Technology	Vesicle Integrity	Cargo Stability	Processing Throughput	Batch-to-Batch Consistency
Ultracentrifugation (UC)	Poor (High risk of rupture)	Moderate (Risk of leakage)	Low (Batch-limited)	Poor (High operator dependency)
Tangential Flow Filtration (TFF)	Excellent (Gentle fluidics)	High (Preserves payload)	Very High (Continuous)	High (Automated parameters)
Size-Exclusion Chromatography (SEC)	Excellent (No shear stress)	Excellent (No degradation)	Moderate (Volume-limited)	Excellent (Highly reproducible)

**Table 5:** Translational challenges and emerging solutions in engineered exosome therapy.

Challenge	Underlying Issue	Impact on Therapy	Current Strategies	Emerging Solutions
Scalability	Low yield, heterogeneity	Poor reproducibility	Ultracentrifugation, precipitation	Bioreactors, continuous production [20,85]
Cargo loading	Inefficient encapsulation	Reduced efficacy	Electroporation, sonication [65]	Programmable RNA loading, endogenous systems [66,85]
Targeting specificity	Off-target uptake	Reduced delivery efficiency	Ligand modification [75,82]	Multivalent targeting, AI-guided design [85]
Biological variability	Cell-source differences	Inconsistent behavior	Standardized culture	Autologous exosomes, improved characterization [18,19]
Safety	Unknown immunogenicity	Clinical risk	Preclinical testing	Immune “stealth” engineering [19]
Regulatory issues	Lack of guidelines	Delayed approval	Case-by-case evaluation	Standardized frameworks [17]

## Data Availability

This is a review article, and all data is included in the manuscript.
